# Rare Side Effect Due to Vedolizumab in a Patient with Ulcerative Colitis: Catatonic Depression

**DOI:** 10.5152/tjg.2025.25210

**Published:** 2025-09-10

**Authors:** Ozan Durmaz, Adnan Ozkahraman, Yusuf Kayar

**Affiliations:** 1 Division of Gastroenterology, Department of Internal Medicine, Van Education and Research Hospital, Van, Türkiye

Dear Editor,

 21-year-old female patient presented with abdominal pain and bloody diarrhea 20 times a day 3 years ago. Colonoscopy was performed and ulcerative colitis with pancolitis was diagnosed, and methylprednisolone, mesalazine tablet, and enema were started. After induction treatment with methylprednisolone, azathiopurine was added to the maintenance treatment because she had more than two attacks per year. However, as she complained of severe drug-induced nausea and vomiting, azathiopurine was discontinued and adalimumab was started. Vedolizumab was initiated due to a lack of clinical, laboratory, and endoscopic improvement after approximately six months of adalimumab treatment. Since the patient’s clinical and laboratory values improved after the first dose, it was planned to continue the treatment. One week after the second dose of vedolizumab, the patient was admitted to the ward with a sudden onset of aggression, one-word speech, suspiciousness, crying episodes, insomnia, and hallucinations. The patient did not have any psychiatric disorders before vedolizumab treatment. On physical examination; fever: 36.5°C, arterial blood pressure: 100/60 mmHg, saturation 98%, respiratory rate: 16 respirations per minute, the patient was uncooperative, agitated, and did not want to be examined, but there was no nuchal rigidity, and other system examinations were found to be normal. No neurologic pathology was considered on examination after neurology consultation. Brain tomography and diffusion magnetic resonance imaging were found to be normal. The patient was evaluated by psychiatry, and haloperidol drops, sertraline 50 mg/day, and olanzapine 5 mg/day were started due to agitation. Although the patient’s complaints of aggression, agitation, and insomnia improved, tremors in hands and feet, complete inability to speak, inability to move, and inability to make eye contact developed. Psychiatric examination revealed that she had no verbal output, did not make eye contact, even if she did, her gaze was very piercing, her affect was markedly blunt, she did not respond to questions, her movements were very slow, her reaction time was prolonged in speech, and she had tremors in her hands and legs. On examination, the cogwheel sign and tremor were found especially in the wrists. Catatonia was considered in the foreground because of mutism, wax elasticity, agitation, stupor, and rigidity. Therefore, diazepam 10 mg/day, sertraline 50 mg/day, and biperiden hydrochloride 2 mg/day were started. After about two weeks under the current treatment, the patient’s complaints improved completely. Considering that the psychiatric condition was due to vedolizumab, it was discontinued, and upadasitinib was started at 45 mg/day. After eight weeks of induction treatment, maintenance treatment with 30 mg/day was continued. The patient did not experience any symptoms or complications after starting upadacitinib treatment. Colonoscopy performed at the sixteenth week of treatment showed that the disease was in complete remission. An informed consent form was obtained from the patient.

Inflammatory bowel diseases (IBD) are multifactorial, chronic, continuous, relapsing, and immune-mediated diseases of the gastrointestinal tract. In recent years, the incidence and prevalence of IBD have increased dramatically in developing countries and the Eastern world, includingTürkiye.^1^^,^^2^ With available effective treatments, treatment goals have been increased from clinical remission to mucosal healing.^3^^,^^4^ Although these therapies have revolutionized the treatment of IBD, serious side effects may rarely develop.^5^

Catatonia is a syndrome characterized by marked impairment in motor, behavioral, and cognitive functions. The etiology of catatonia is multifactorial and may be associated with many factors such as infections, metabolic disorders, neurological diseases and medications.^6^ Catatonia is generally associated with imbalances in the dopaminergic and GABAergic (inhibitory pathways in the central nervous system) pathways. Although the effect of vedolizumab on these systems is not fully known, it may have caused catatonia by affecting the neurotransmitter balance in the central nervous system (CNS) via the gut-brain axis. In this case, neurologic and metabolic causes have been excluded, and this case, triggered by vedolizumab, is presented. In the literature, neuropsychiatric side effects associated with anti-TNF agents are better documented. Anti-TNF agents reduce inflammation by blocking the action of TNF-α (Tumor Necrosis Factor alpha). However, TNF-α plays an important role not only in peripheral inflammation but also in the regulation of neuroinflammation in the CNS. TNF-α is a critical molecule in the regulation of synaptic plasticity, neurotransmitter release, and neuroinflammation in the CNS.^5^ Anti-TNF therapies may lead to neuropsychiatric symptoms by affecting these mechanisms. In the literature, depression, anxiety, psychotic disorders, and even suicide attempts have been reported in patients receiving anti-TNF treatment.^5^ Nevertheless, neuropsychiatric side effects associated with vedolizumab have rarely been reported. This is explained by the fact that vedolizumab has fewer systemic effects. Vedolizumab is an anti-integrin agent that inhibits gut-specific lymphocyte migration. Although the risk of systemic immunosuppression is thought to be low due to its selective effect, neurologic side effects of integrin antagonists are usually associated with immunosuppression and alteration of inflammatory processes in the CNS and are rarely reported in the literature. These side effects include progressive multifocal leukoencephalopathy (PML), headache, dizziness, seizures, and other neurological disorders. Progressive multifocal leukoencephalopathy, which can result in progressive neurological disorders, cognitive decline, motor dysfunction, and death, usually develops when integrin antagonists such as Natalizumab block the entry of immune cells into the CNS, leading to reactivation of the JC (John Cunningham virus) virus.^6^

Although the mechanisms of neuropsychiatric side effects associated with vedolizumab therapies are not fully understood, several hypotheses have been proposed.^5^^,^^7^ The gut-microbiota-brain axis is a concept that explains the effects of gut microbiota on the CNS. The gut microbiota can affect the production of neurotransmitters (e.g., serotonin, dopamine) in the CNS and regulate the release of inflammatory cytokines. Vedolizumab may have caused neuropsychiatric symptoms through this axis by affecting the gut microbiota.^5^^,^^7^

In conclusion, this case demonstrates that an intestine-specific integrin antagonist such as vedolizumab may cause similar neuropsychiatric side effects. Mild neurologic side effects are usually seen early in treatment and diminish over time. But, in rare cases, serious side effects may develop. This case shows that vedolizumab may cause serious neuropsychiatric side effects such as catatonia by affecting the neurotransmitter balance in the CNS, and its implications for clinical practice should be carefully evaluated.

## Data Availability

The data that support the findings of this study are available on request from the corresponding author.
